# GLP 1 Regulated Intestinal Cell’s Insulin Expression and Selfadaptation before the Onset of Type 2 Diabetes

**DOI:** 10.15171/apb.2019.039

**Published:** 2019-06-01

**Authors:** Shivani Srivastava, Harsh Pandey, Surya Kumar Singh, Yamini Bhusan Tripathi

**Affiliations:** ^1^Department of Medicinal Chemistry, Institute of Medical Sciences, Banaras Hindu University, Varanasi, U.P, India.; ^2^Department of Endocrinology and Metabolism, Institute of Medical Sciences, Banaras Hindu University, Varanasi, U.P, India.

**Keywords:** Glucagon like peptide 1, Type 2 Diabetes, Liraglutide, Insulin, Intestine, Apoptosis

## Abstract

***Purpose:*** Basically insulin is known to be secreted by β cells of the pancreas. Recently, it has also been found to be produced and expressed by intestinal epithelial cells with the help of L cells secreting glucagon like peptide 1 (GLP 1). Here, we have studied the same intestinal insulin expression property in T2D rats.

***Methods:*** Following 2 weeks of high fat diet (HFD) consumption, we have been given a single dose of streptozotocin (STZ) (35 mg/kg bw). Rats were then sacrificed after 1, 7 and 21 days. The GLP 1 analogue, liraglutide was also given to one group of diabetic rats, upto their respective durations. Intestinal cells apoptosis were checked by tunnel assay, Incretin hormones secretion and dipeptidyl peptidase 4 (DPP-IV) activity were analyzed through ELISA and immunohistochemistry was used to determine the insulin expression of intestine at different time interval during diabetes progression.

***Results:*** As compared to 1 and 21 days, we have found minor cells apoptosis in 7 days group along with high level of GLP 1 in diabetic model. Further, these effects were enhanced by liraglutide. In response to these we have found, decreased insulin expression after 21 days and with no significant effect upto 7 days in diabetic control groups. In contrast to this, GLP-1 level and insulin expression enhances prominently after 7 days of liraglutide treatment.

***Conclusion:*** These results explain the self-adapting approach of intestinal cells against diabetes onset and insulin expression enhancing property of liraglutide under stressful conditions. This study should be continued in future for the development of intestinal insulin producing drugs, to control diabetes under irreversible β cells damage.

## Background

Intestine, as we know is considered as the second brain of the body. Being the most prominent organ, it influences physiology of other organs also. Its secretion, absorption, digestion, resorption etc maintains the whole organism’s cellular and molecular mechanisms. Our daily life style of diet somewhere decides the way of the growth of our body, mind and general health, which directly interacts with gastrointestinal tract (GIT). Thus, any inflammation, disorder or diseases of the intestine could be fatal for the whole organism.

Diabetes is on regular rise worldwide. It is due to progressive β cells damage. Intestinal function and incretin secretion are reported to directly regulate the health of the pancreas. Glucagon like peptide 1 (GLP 1) and g*lucose*-dependent insulinotropic polypeptide (GIP) are the two primary incretin hormones, which regulates glucose induced insulin secretion from pancreatic β cells. Their physiological roles are governed by the activity of an intestinal proteolytic enzyme dipeptidyl peptidase 4 (DPP-IV) and through regulation of GLP-1 and GIP receptors on pancreas.^1^

Recent reports proved that, intestinal epithelial cells have the potential to get converted into insulin producing cells.^2-4^ One of the incretin hormone, GLP 1 (1-37) has been studied to act as an inducible factor for the expression and production of insulin in intestinal epithelial cells.^3^ Studies suggested that, the mechanism of pancreatic and intestinal endocrine-lineage cells fate determination is commonly controlled by the Notch signalling pathway. Thus, by controlling the genes and expressions responsible for pancreatic endocrine differentiation, GLP 1 (1-37) converts epithelial progenitors of small intestine into insulin-producing cells.^3^ Besides the anatomical and physiological function of GIT, its microbiota is equally important in governing multiple functions. A human commensal bacteria have been engineered to secrete GLP 1 (1-37), able to reprogram human and rat intestinal epithelial cells to glucose responsive insulin secreting cells expressing the β cells markers such as MafA, PDX 1 and forkhead box protein A2, in order to treat diabetes.^2,5^

Earlier, we reported the self-adaptive mechanism of β-cells through 10 days expression kinetics against streptozotocin (STZ) induced stress. In that study, β cells showed the self-adaptation by compensating the starvation-induced autophagy, inflammation, hypoxia and disturbed immunity, via modulating all the expressions needed for β cells damage and survive. Overall, β cells balances the pathways, including apoptosis, immunity, neo-genesis, autophagy and proliferation, in order to save the cellular energy.^6^

This is our hypothesis that the intestinal cells transiently produce insulin with the help of GLP 1 as an adaptive mechanism, against stress before the onset of diabetes. Here, we have accessed the kinetic study to reveal the pattern of apoptosis as well as insulin expression and incretin hormones secretions from intestinal cells at different time interval, during the progression of type 2 diabetes and also judged the insulinotropic potential of GLP 1 analogue liraglutide under the same conditions.

## Materials and Methods

For preparation of high fat diet (HFD); normal diet, lard, casein, cholesterol, vitamin & mineral mix, DL-methionine, yeast powder and sodium chloride were used. The following materials were used for immunohistochemistry: mouse monoclonal anti-insulin antibody (HB125-BioGenex), anti-mouse-AF488 (Invitrogen, USA) (Green) secondary antibody. Streptozotocin from Sigma Aldrich. In addition, Leica RM2125 RT rotator microtome (Leica Bio systems Nussloch GmbH, Nussloch, Germany) was used.

### Design of work

The Charles foster rats were procured from the central animal facility of the institute and randomly divided into the following groups:

1. Normal Control: kept on normal diet.
2. Diabetic control: After 2 weeks HFD (contains as a percentage of total kilocalorie; 58% fat, 25% protein and 17% carbohydrate),^7^ rats were fasted overnight. Next day, the injection of STZ was given at the dose of 35 mg/kg bw along with liraglutide (1.2 mg/kg bw) to one out of two divided groups. In both groups, the consumption of HFD was stopped 8 hours, before the injection of STZ. The type 2 diabetes validity was checked via HOMA IR and Insulin tolerance test with 0.5 U/ kg bw Insulin injection (data not shown). The animals were then sacrificed after 1, 7 and 21 days of STZ injection. The blood was collected from brachial artery and jejunum part of the small intestine was dissected out for further studies.

### Tunnel assay

Apoptosis detection was done using “TACS^®^ 2 TdT Fluorescein Kit - Trevigen”.

### Biochemical assays on plasma

The levels of GLP-1 and GIP in plasma were measured using enzyme immunoassay (EIA) kits (Sigma Aldrich- RAB0201 and RAB0209), and plasma DPP IV activity was measured using a fluorimetric assay kit (Sigma Aldrich- MAK088) as per manufacturer’s instructions.

### Immunohistochemistry

Ten minutes xylene treatment was given to the paraffin sections of pancreas in order to remove the paraffin. Through 90%, 70% alcohol and water, the sections were rehydrated for 5 min. each. The process of antigen retrieval was done by putting the citrate buffer dipped slides in EZ Retrieval System V.3 (Bio Genex). Then after sections were washed with citrate buffer twice and also two times for 10 minutes each with 1X PBS (130 mM NaCl, 7 mM Na_2_HPO_4_, 3 mM KH_2_PO_4_, pH 7.4), following the above all sections were incubated for 2 hours at room temperature with blocking solutions [0.1% Triton X-100, 0.1% BSA, 10% FCS, 0.1% sodium deoxycholate and 0.02% Thiomersal (an anti-fungal agent), in 1X Phosphate Buffered Saline (PBS)]. All slides were transferred in primary antibodies, for overnight at 4^o^C. Next day, slides were washed three times with PBST (0.1% Triton X in 1X PBS) for 10 minutes each. After washing, each section was passed through 2 hours incubation with secondary antibody at room temperature. Before staining with DAPI (1 µg/mL DAPI in 1X PBS), all sections were washed 3 times for 10 minutes each, in PBS with Tween 20 (PBST). After 30 minutes incubation with DAPI, slides were mounted in DABCO and examined under Zeiss LSM510 Meta confocal microscope. Image analysis was done by using Zen Black (2012) software.

### Statistical analysis

One-way ANOVA test followed by post hoc analysis with Dunnett’s test was done for each experiment. All results were expressed as means ± SD. Statistical significance was taken at *P* ≤ 0.05.

## Results and Discussion

### Apoptosis of intestinal cells

**Figure 1:**
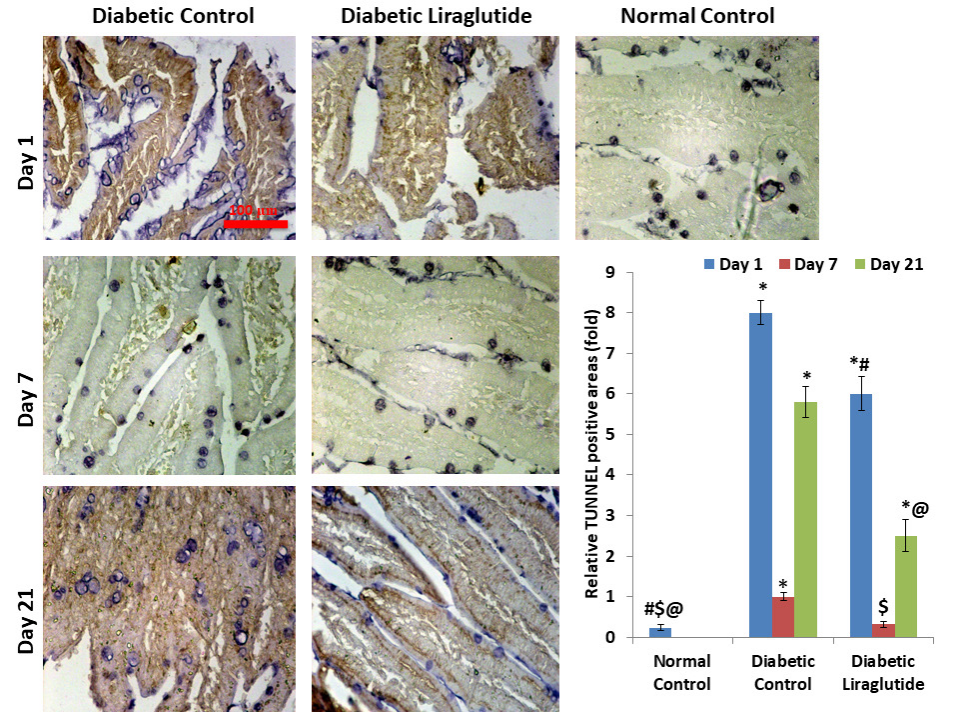
Tunnel assay. The apoptosis were down regulated in 7 days group of both diabetic control and diabetic liraglutide, indicating the self-adaptation of intestine towards stress as well as the co-stimulatory effect of liraglutide. The image was taken at 40X magnification. Scale bar 100 µm. Each value represents the mean ± SD (n=6); *P < 0.05, compared with normal control, # *P* < 0.05, compared with day 1 diabetic control, $ *P* < 0.05, compared with day 7 diabetic control, @ *P* < 0.05, compared with day 21 diabetic control.

The viability of intestinal cells gets lost after 1 day of STZ injection following 2 weeks of HFD consumption. Here, in 7 days, the intestine adapts to the stress most probably by self-proliferation. In 21 days, the cells again lost their viability. Liraglutide augmented this self-adaptation process, throughout the experiment. (Figure 1).

### Plasma GLP 1, GIP and DPP IV activity

DPP-IV activity was exponentially enhanced in a time dependent manner in case of diabetic controls. But its activity was reduced significantly in case of liraglutide treatment except in case of day 7.

As compared to the normal group, the GLP-1 level was significantly reduced throughout the experiment in the diabetic control groups. This level was upregulated by liraglutide treatments. In contrast to these changes, there was an extensive increase of GIP in diabetic control after one day of STZ treatment, but then it gradually decreased upto 21 days. In this case, we have found some interesting pattern of enzyme and hormonal response. Here, in the group of 7 days, the activity of DPP-IV and the level of both GLP-1 & GIP gets enhanced as compared to the group of both 1 and 21 days, in case of liraglutide treated rats. Interestingly, the same pattern was also found with GLP 1 in a group of 7 days diabetic control.

Liraglutide is a GLP 1 analogue and also known as a GLP 1R agonist. According to our results, it enhances the GLP 1 at day 1, 7 and 21. But this change is not consistent for GIP level in plasma in day 1 group, its excessive secretion also suggests the self-protective response of intestinal cells in the diabetic control group. It indicates, another mode of action with respect to GIP. Liraglutide has already been known to partially inhibit the DPP-IV and alters its activity in the taste buds of the circumvallate papillae in type 2 diabetic rats. Liraglutide upregulated the expressions of DPP-IV in taste buds and downregulated the same in the hypothalamus of T2DM rats.^8^ Our study has also shown the effect of Liraglutide on DPP-IV activity. It significantly reduces the activity of DPP-IV in plasma of day 1 and day 21 group. Nonsignificant effect on day 7 is interestingly an important point of consideration.

**Figure 2:**
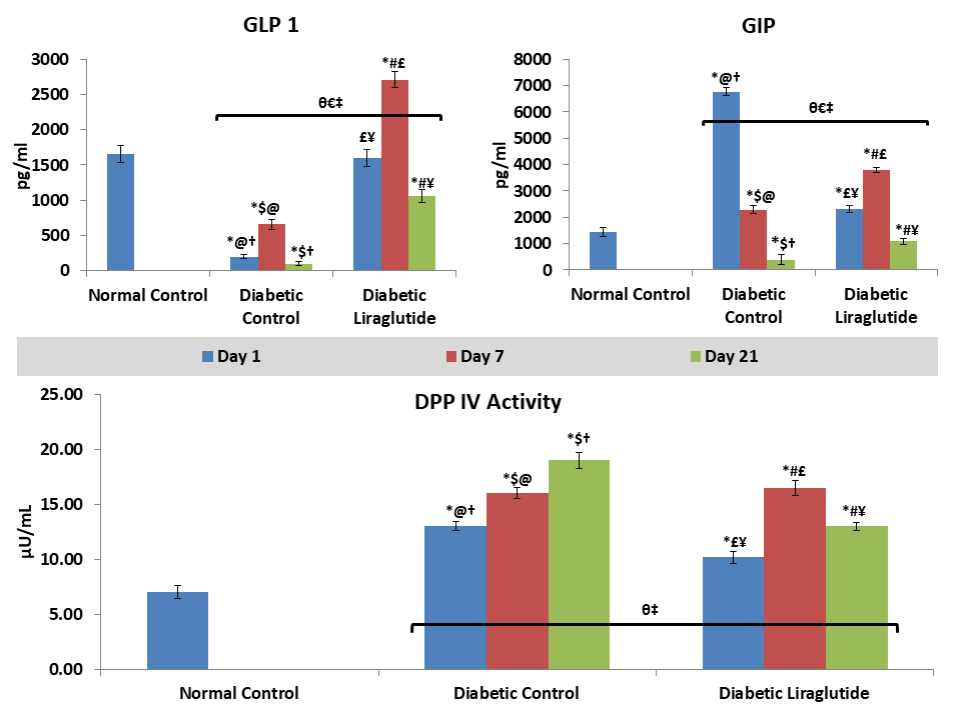
Plasma level of GLP-1, GIP and DPP-IV enzyme. Data shows the self-adaptive response of intestinal cells in the diabetic control group after 7 days through secretion of more GLP 1. Furthermore, liraglutide enhanced the secretion of GLP-1 and also modulates the secretion of GIP and DPP-IV activity. The protective action of GIP works intensively after 24 hours in diabetic control group. Each value represents the mean ± SD (n=6). *P < 0.05 compared with Normal Control, $*P* < 0.05 comparison of day 1 diabetic control with days 7 & 21 diabetic control , †*P* < 0.05 001 comparison of day 7 diabetic control with day 1 & 21 diabetic control, @*P* < 0.05 comparison of days 21 diabetic control with days 1 & 7 diabetic control, #*P* < 0.05 comparison of day 1 diabetic liraglutide with days 7 & 21 diabetic liraglutide, ¥*P* < 0.05 001 comparison of day 7 diabetic liraglutide with day 1 & 21 diabetic liraglutide, £*P* < 0.05 comparison of days 21 diabetic liraglutide with days 1 & 7 diabetic liraglutide, θ*P* < 0.05 comparison of day 1 diabetic control with days 1 diabetic liraglutide, €*P* < 0.05 comparison of day 7 diabetic control with days 7 diabetic liraglutide, ‡*P* < 0.05 comparison of day 21 diabetic control with days 21 diabetic liraglutide.

These results explain the additional mode of action of Liraglutide beyond the GLP-1R agonism (Figure 2).

### Insulin expression in intestine

**Figure 3:**
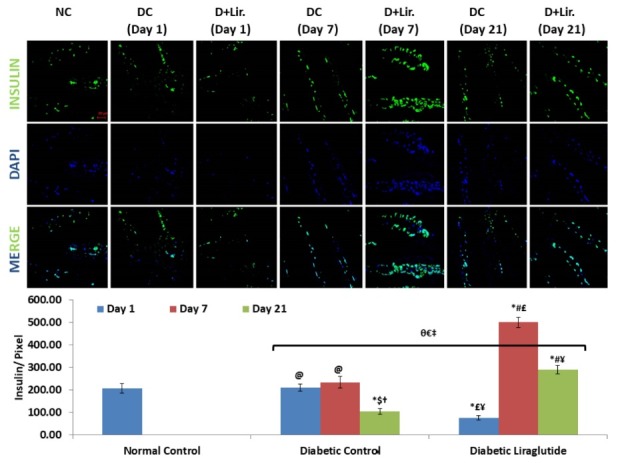
Confocal Imaging of Insulin. The intestine of diabetic control groups adapts themselves by enhancing the insulinotropic effect upto 7 days, non-significant to normal control, in order to overcome the excessive burden of stress, while liraglutide co-stimulate this effect, more specifically after 7 days. Each value represents the mean ± SD (n=6). The expression was merged with DAPI (blue). The image was taken at 40× magnification. Scale bar 20 µm. The intensity was measured in pixel values. *P < 0.05 compared with Normal Control, $*P* < 0.05 comparison of day 1 diabetic control with days 7 & 21 diabetic control , †*P* < 0.05 001 comparison of day 7 diabetic control with day 1 & 21 diabetic control, @*P* < 0.05 comparison of days 21 diabetic control with days 1 & 7 diabetic control, #*P* < 0.05 comparison of day 1 diabetic liraglutide with days 7 & 21 diabetic liraglutide, ¥*P* < 0.05 001 comparison of day 7 diabetic liraglutide with day 1 & 21 diabetic liraglutide, £*P* < 0.05 comparison of days 21 diabetic liraglutide with days 1 & 7 diabetic liraglutide, θ*P* < 0.05 comparison of day 1 diabetic control with days 1 diabetic liraglutide, €*P* < 0.05 comparison of day 7 diabetic control with days 7 diabetic liraglutide, ‡*P* < 0.05 comparison of day 21 diabetic control with days 21 diabetic liraglutide.

As compared to normal control, the expression of insulin upto 7 days was not significant, but gets down regulated after 21 days, in case of diabetic control. Interestingly, after 1 day, insulin expression after a single dose of liraglutide gets reduced. It is assumed that STZ injection after 2 weeks of HFD consumption suddenly enhances the stress conditions in 24 hours, and this effect was counteracted through the intestine itself by activating insulin expression non-significant to normal control. Here, intestinal cells self-adapt themselves most probably by proliferating its epithelial cells and converting its epithelial cells into insulin expressing cells. This mechanism was not required in case of liraglutide treatment because of its antioxidant properties,^9,10^ leading to physiologically lower level of free radicals as compared to diabetic control needed to enhance insulin expression by epithelial cells within 24 hours after STZ injection. In contrast to this, the self-adapting effect of intestine continues upto 7 days and gets downregulated after 21 days of STZ. Interestingly, in case of liraglutide, the expression of insulin gets prominently enhanced after 7 days following its reduced expression after 1 day of STZ, and gets reduced again after 21 days, significantly higher than normal and diabetic controls. These results, indicates the co-stimulatory insulinotropic effect through liraglutide after 7 days. Thus, showed the adaptive approach taken by intestinal cells upto 7 days after STZ injection and co-stimulatory insulin enhancing effect of liraglutide (Figure 3).

These studies significantly prove the self-adapting approach of the intestine before the onset of diabetes, as indicated by the insulin expression, reduced apoptosis and elevation of incretin hormones, required to counter the alternative stressful microenvironment. Here, STZ induced stress was overcome by incretin hormones i.e., GIP after 1 day and GLP 1 after 7 days. Liraglutide, on the other hand upregulate this adaptive GLP 1 secretion and reduces apoptotic response. In addition to this, liraglutide also modulates the secretion of GIP and DPP-IV. The liraglutide significantly inhibits the DPP-IV activity in 1 and 21 days group and enhances GIP level in 7 and 21 days group. This interesting DPP-IV inhibitor as well as GIP enhancing properties of liraglutide should be studied deeply in the future, in order to reveal the mechanism of action beyond GLP 1 regulated physiological actions. As liraglutide was unable to inhibit the activity of DPP IV in day 7 group, might concludes the excessive stress at this stage, which was beyond the physiological potential of liraglutide. Insulin secretion from insulin producing epithelial cells of the intestine was studied to occur at a physiologically appropriate glucose level.^3^ This means, the insulin secretion from intestine depends upon the physiological range of stress induced by hyperglycemia. Thus, within 7 days, the burden of stresses was at its peak, that were compensated through the intestinal insulin producing epithelial cells proliferation and GLP 1 secretion.

Liraglutide, the GLP-1 analogue has been studied for its role in β cells proliferation through Akt/FoxO1/p27 and protective action through PI3K/Akt/FoxO1 pathways.^11,12^ Studies had also shown that ablation of Fox O1 gene leads to the generation of functional insulin-producing cells in the gut.^4,13^ Through above studies, it could be the common mechanism through which liraglutide proliferate intestinal epithelial cells and enhanced the expression of insulin.

This study has many lacunas, including deep analysis of stress and proliferative markers, the individual effects of HFD and STZ induced intestinal damage, as well as the histopathological changes. But, still have provided the significant mechanism of intestinal self-adaptation during type 2 diabetes progression and this information will provide great help in future for intestinal insulin producing drugs development in case of unresponsive behavior of β cells for their specific medicines or their decreased number in the condition of severe diabetes.

## Conclusion

These studies have provided a new therapeutic mechanism against diabetes, beyond specific treatment approach for β cells of the pancreas. Reveals the new pathway of insulin secretion through the intestine in addition to the pancreas. Thus, could provide the new drugs which will enhance the GLP 1 mediated conversion of intestinal epithelial cells into insulin positive cells. Further studies should be done to describe the gut-pancreas axis rhythmic communication at the time of stress.

## Ethics Issues

The protocol was approved by the Institute Ethical Committee (Dean/2015/CAEC/1266), Institute of Medical Sciences, Banaras Hindu University.

## Conflict of Interest

The authors, ethical committee, and funding agencies declared no conflict of interest.

## Acknowledgments

We are thankful to Prof. Shail Kumar Chaube and his students, Department of Zoology, Institute of Sciences, BHU, for their help in apoptosis assay. Heartiest thanks being given to ISLS, Institute of Sciences, BHU for providing us the facility of Confocal Microscopy.
